# A deep learning-based approach for detecting anomalous behavior in safety-critical spaces

**DOI:** 10.3389/frai.2026.1702756

**Published:** 2026-03-31

**Authors:** Aqib Anees, Syed Asim Jalal, Hassan Jalil Hadi, Naveed Ahmad, Mohamad Ladan

**Affiliations:** 1 Department of Computer Science, University of Peshawar, Peshawar, Pakistan; 2 CEMSE Division, King Abdullah University of Science and Technology, Thuwal, Saudi Arabia; 3 College of Computer and Information Sciences, Prince Sultan University, Riyadh, Saudi Arabia

**Keywords:** AI, deep learning, road crash accident, safety, YOLO

## Abstract

Wrong-turn violations in safety-critical spaces such as road roundabouts are a type of traffic violation that can lead to traffic congestion and increase the risk of road crashes. Although many researchers have focused on detecting various traffic violations, wrong-turn violations have not received enough attention. This may be due to a lack of relevant datasets. This study aims to address this gap. We developed a deep learning–based approach to detect wrong-turn traffic violations at roundabouts. The proposed system captures video from strategically placed cameras at roundabouts, which is then fed into an artificial intelligence (AI) model capable of detecting vehicles committing wrong-turn violations in real time. For this purpose, we utilized the popular You Only Look Once (YOLO) algorithm. Due to the absence of an existing dataset for this specific type of violation, we created our own. Images were collected and annotated from local roundabouts in Peshawar, Pakistan. The YOLO model was trained on this dataset and evaluated using standard performance metrics, including accuracy and recall. The results suggest that the proposed approach has strong potential for refinement and real-world implementation.

## 1 Introduction

Vehicles play an essential role in modern life, and imagining a world without them is difficult. The increased affordability of vehicles worldwide has led to many challenges, including road safety issues due to traffic rule violations. Enforcing traffic laws has become extremely critical to ensure road safety for all stakeholders. According to the World Health Organization (WHO), road crashes cost countries up to 3% of their GDP (WHO, 2023), highlighting the significant economic impact aside from personal and societal losses. In 2023, 4,241 road crashes were reported in the Khyber Pakhtunkhwa province of Pakistan alone, resulting in numerous injuries and fatalities (PBS, 2023). Common traffic violations include speeding, running red lights, using mobile phones while driving, violating lane regulations, reckless driving, and driving in the wrong direction. Modern technologies are increasingly being used to detect these violations and have become valuable tools for law enforcement agencies. Sensors, radars, and cameras are among the key technologies deployed for this purpose. Artificial Intelligence (AI) is also being applied to detect traffic violations. However, wrong-turn violations at roundabouts remain understudied in current technology-driven research. One possible reason for this gap is the lack of relevant datasets. This study aims to address this gap by proposing an AI-based approach to detect wrong-turn violations at roundabouts.

### 1.1 Traffic violations at roundabouts

**Figure 1:**
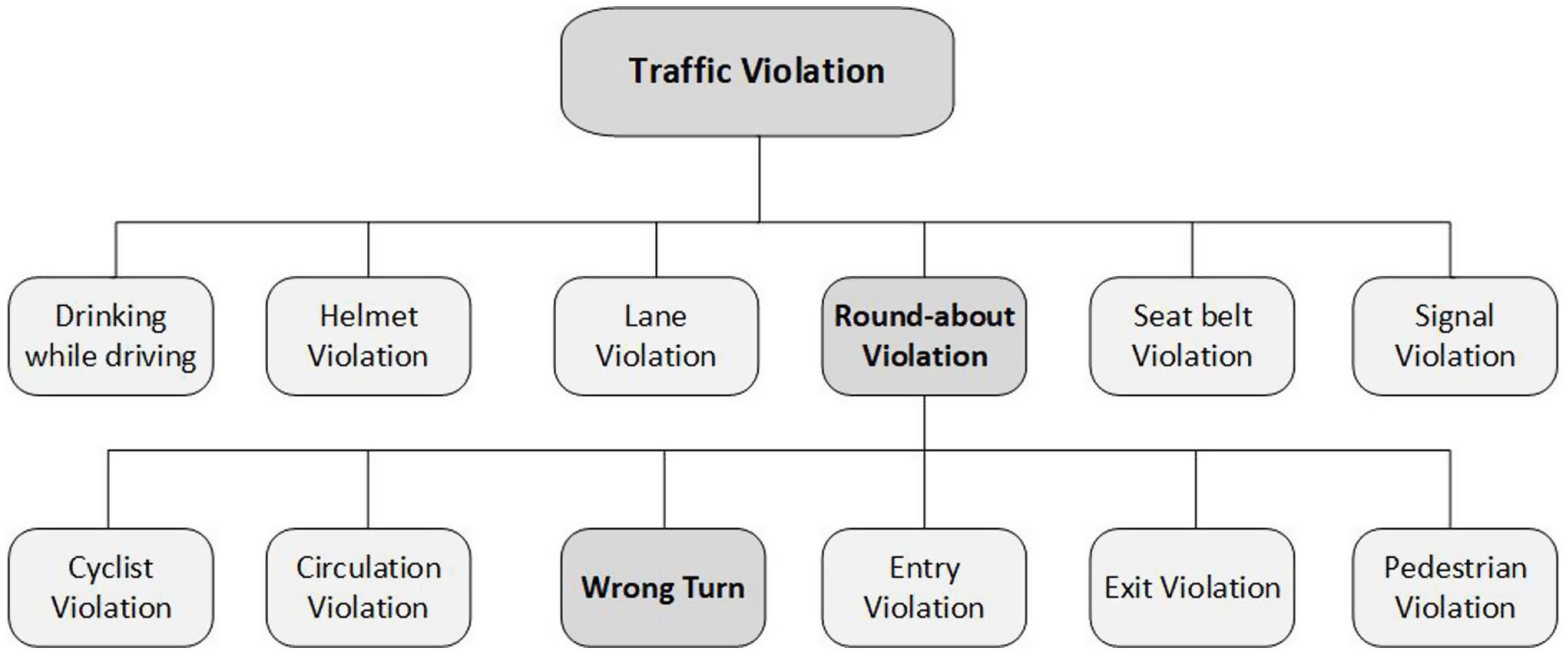
Taxonomy of traffic violations.

Figure 1 presents a classification of common traffic violations, including wrong-turn violations at roundabouts. Other common violations include signal violations (Anand et al., 2021), lane violations (Arnob et al., 2020), seat belt violations, and helmet violations for motorcyclists (Afzal et al., 2021; Maduri et al., 2021). Violations at roundabouts (Hourdos et al., 2014) can lead to driver confusion and significantly increase the risk of crashes, (Obaid and Mohammed, 2024; Rassokha et al., 2021). After detecting the ROI, a map of these regions is created for further analysis (Google, 2019). Common traffic violations include speeding, running red lights, using mobile phones while driving, violating lane regulations, reckless driving, and driving in the wrong direction (Sloan, 2020).

Technology-based methods such as machine learning and object tracking systems have been widely used in various research studies to detect traffic violations. Recently, deep learning algorithms such as You Only Look Once (YOLO) and centroid tracking have been employed for real-time violation detection (Reddy et al., 2021; Bedruz et al., 2018). Other notable efforts include the detection of wrong-way driving (Zhu and Yan, 2022), red light violations (Rahman et al., 2020), and helmet use (Afzal et al., 2021). Even robotic systems have been introduced to monitor roads and identify infractions (Wang et al., 2023). Deep learning approaches have shown higher accuracy in detecting various types of road violations (Xu et al., 2021; Azimjonov et al., 2023; Rahman et al., 2020).

**Figure 2:**
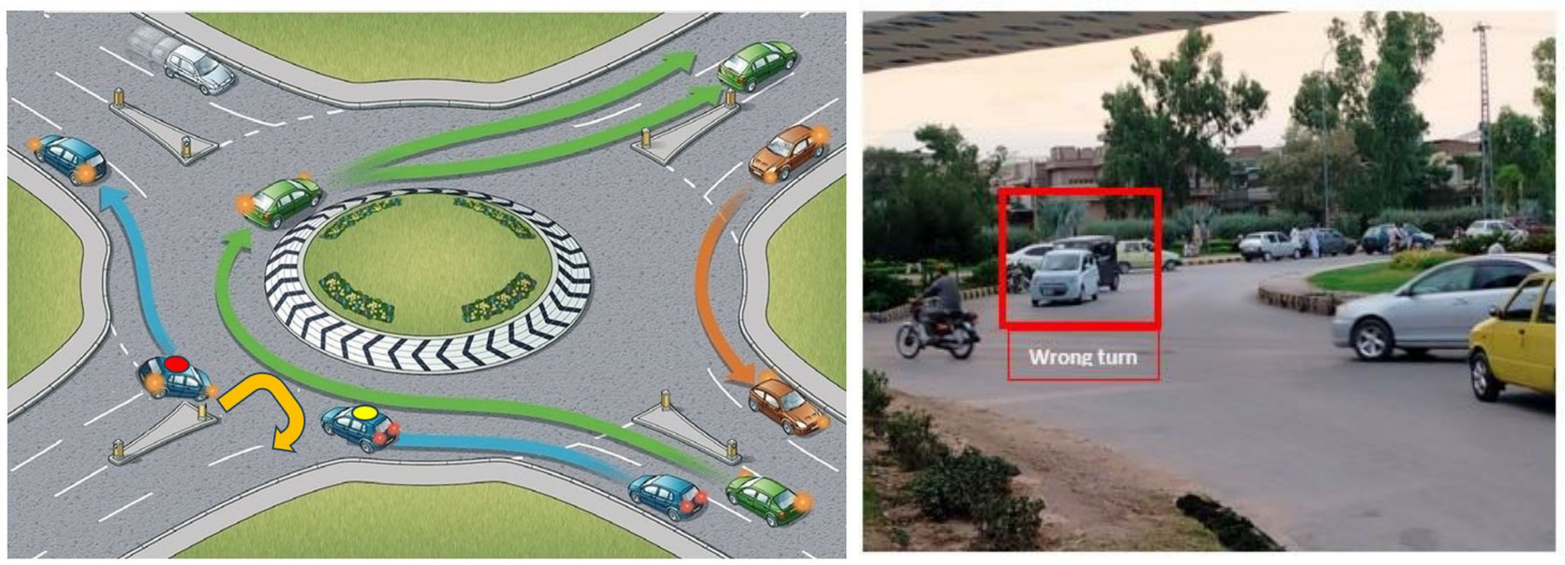
Example of a wrong turn at a roundabout (Zutobi Drivers Ed, 2024) and an example of a real-life roundabout violation.

**Figure 3:**
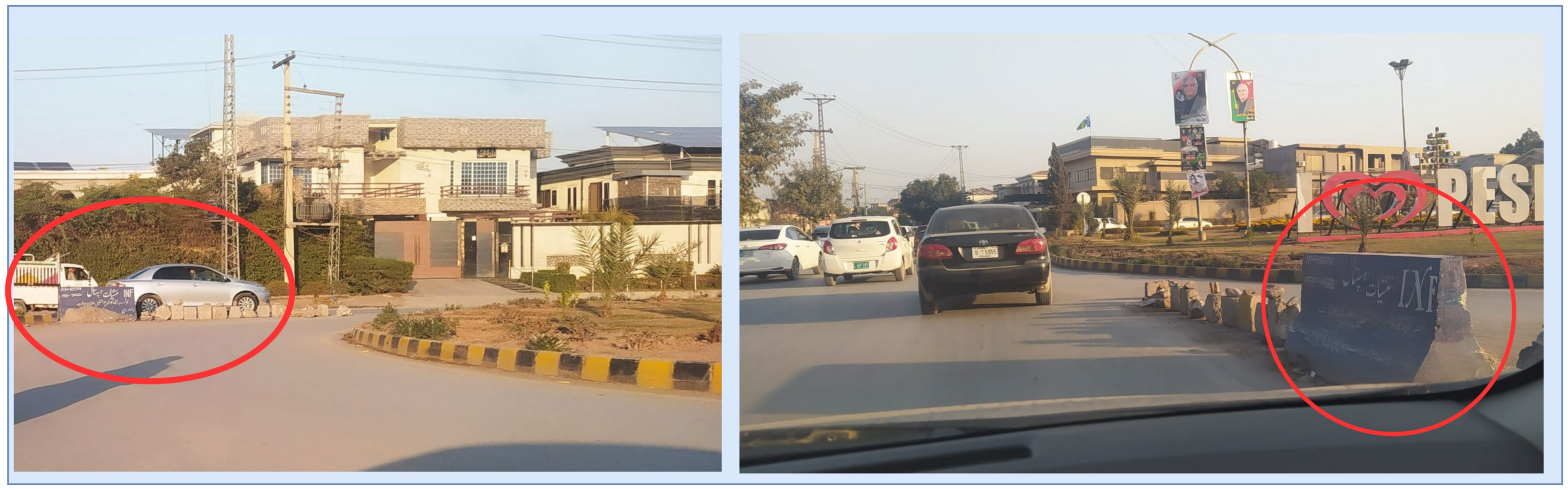
Run-of-the-mill solutions to wrong-turn violations.

A roundabout is a circular road structure designed to improve traffic flow. These intersections do not use traffic signals or stop signs. They rely on signs and geometric designs to manage vehicle movement. Roundabouts typically include a central island that slows down traffic. It ensures that vehicles travel in a clockwise (or counterclockwise in some regions) direction. Drivers must yield to traffic already in the roundabout before merging into the appropriate lane to exit the roundabout. Despite their usefulness, roundabouts are not immune to violations by drivers, which can take several forms: Entry violations occur when drivers do not yield to circulating traffic. This happens often due to misjudging speed or space, leading to collisions or delays. Exit violations occur when drivers do not use the correct lane or fail to signal while exiting, causing confusion and potential crashes. Circulation violations involve illegal lane changes, stopping inside the roundabout, or failing to yield to vehicles already in the roundabout. Wrong-turn violations occur when drivers enter the roundabout against the traffic flow in the wrong direction to take a shortcut or a U-turn without following the complete circular path. This is clearly a dangerous behavior and increases the risk of crashes. Figure 2 illustrates a wrong-turn violation and also provides a real-life example of such a violation. In some places, traffic law enforcement agencies use physical barriers at roundabouts to prevent such violations, as shown in Figure 3.

The above examples highlight the need to detect wrong-turn violations effectively, an underexplored issue within the broader research area of traffic violation detection. A key challenge in this area is the absence of a dedicated dataset, which hinders the development and testing of AI-based models for this purpose. This study proposes the use of deep learning, specifically the YOLO (You Only Look Once) algorithm, for detecting wrong-turn violations. The proposed approach involves deploying strategically placed cameras for detecting these violations. Although detection alone can be performed using a single overhead camera, this setup does not provide a mechanism to identify the vehicle’s number plates, which is necessary for a complete enforcement system.

### 1.2 Significance of the study

This study is significant for several reasons. First, it highlights a gap in existing traffic violation detection research related to this specific violation. Second, it introduces a newly created dataset tailored to these wrong-turn violations. Third, a deep learning based approach is proposed to detect wrong-turn violations at roundabouts.

### 1.3 Contributions

This research contributes to the field of AI-based traffic violation detection in the following ways:

1. Proposes a deep learning-based approach for detecting wrong-turn traffic violations at roundabouts
2. Develops a custom image dataset specifically containing wrong-turn violations, suitable for training deep learning models
3. Evaluates the proposed approach using standard deep learning performance metrics

### 1.4 Article organization

The remainder of this article is structured as follows: Section 1 discusses traffic violations at roundabouts, with a focus on wrong-turn violations, and outlines the research objectives. Section 2 reviews deep learning methods and their applications in traffic surveillance. Section 3 describes the proposed methodology, including data collection, annotation procedures, and workflow diagrams. Section 4 presents data analysis and discusses the effectiveness of the proposed model. Section 5 concludes the study and discusses its implications for traffic management.

## 2 Related research

Several research projects have focused on detecting traffic violations using different approaches and techniques, including AI, sensors, and other electronic monitoring systems.

### 2.1 Technology-based traffic violation detection

Anand et al. (2021) proposed an ultra- wideband (UWB) vehicular radar method for the detection of two-wheeled vehicles and pedestrians. In this approach, the Doppler is estimated using the echo’s trajectory. The trajectory estimate, combined with the Hough transform and strong clutter discrimination, is used to identify pedestrians and two-wheelers. While their work does not clearly target any violations, this type of vehicle detection can contribute to monitoring certain violations.

Reddy et al. (2021) and Zhu and Yan (2022) proposed systems capable of detecting red signal violations and alerting offenders to penalties. They suggested that traffic violation detectors can identify signal violations using computer vision. The proposed methods use computer vision to detect common traffic violations in real time with high accuracy. Although our objectives in this study are similar in the sense that we use a camera for violation detection, the key difference lies in the type of violation: We specifically focus on wrong-turn violations at roundabouts, using video streams.

Rahman et al. (2020) proposed a system for wrong-way vehicle detection in real time using the you only look once (YOLO) object detection and centroid tracking algorithms. The system first detects all vehicles in the input using the YOLO algorithm, which generates bounding boxes for each vehicle. Then, the centroid tracking algorithm follows each vehicle in a specified area of interest. By calculating the centroid height of each vehicle across consecutive frames, the direction of the motor can be determined. The system demonstrated promising results when tested on traffic videos. The accuracy of the system depends on the performance of the YOLO detection. The system can also store an image of the vehicle if it is on the wrong side. A limitation of this system lies in the centroid tracking algorithm, where the centroids of the object must be placed together between consecutive frames or it might lose the ID number.

The approach proposed by Afzal et al. (2021) uses a Faster Region-Based Convolutional Neural Network (R-CNN) model of deep learning that has two phases. A Region Proposal Network (RPN) is used in the first phase to find the helmet and in the second phase to identify the found helmet. The authors collected their own dataset from real-world scenarios in Pakistan, which included various challenges such as blurry images, images of people wearing hats, low-resolution images, images captured from the front, side, and rear, occlusions, and images of people holding helmets. The Faster R-CNN model was then tested using this dataset, and the researchers were able to outperform previous techniques in terms of accuracy and efficiency. The authors also discussed the limitations of their approach and suggested several avenues for future research, such as producing more datasets. Our suggested solution for roundabouts is based on the use of the YOLO algorithm to detect violations. It involves two steps: In the first step, it detects vehicle(s), and in the second step, it determines their status—whether they are obeying or violating the traffic rules.

### 2.2 AI-based traffic violation detection

Wang et al. (2023) and Agrawal et al. (2017) proposed a method using the YOLO object detection algorithm for identifying automobiles traveling in the wrong direction. Vehicle boundary boxes are established, and a centroid-based technique is used for direction detection. The Euclidean distance between the new and old centers is determined using a centroid tracking method to track the vehicles. However, a potential drawback of this method is that overlapping objects can lead to vehicle ID switching. In this proposed system, vehicles are detected based on bounding boxes, with a focus on wrong turns at roundabouts rather than regular roads or vehicle direction (Agrawal et al., 2017).

Mandal and Adu-Gyamfi (2020) conducted comparative studies of several deep learning object detectors combined with the Deep Simple Online and Realtime Tracking (Deep SORT) algorithm. They showed that YOLOv4, Deep SORT, CenterNet, and their combinations performed best for counting all vehicles on the road. They also suggested that these combinations may be further optimized for improved tracking and condition detection. To illustrate their findings, they used a methodology that involved evaluating each model combination on 546 one-min video segments. Certain models produced very high false-positive and false-negative rates in their predictions. Combining CenterNet with Deep SORT yielded an average accuracy of 95.9%, followed by Detectron2 at 92.5% and YOLOv4 combined with Deep SORT at 90.9%.

Usmankhujaev et al. (2020) proposed a real-time closed-circuit television (CCTV) approach for detecting cars driving in the wrong direction on the road. Their method involves three validation phases: YOLOv3 detection, Kalman filter tracking, and the Entry–Exit approach. After evaluating multiple models and algorithms for both the tracking and detection components for their assignment, they selected the most effective algorithm. To implement the Entry–Exit validation technique, the author had to manually identify zones considered appropriate or inappropriate for entry or exit. Their approach achieved an accuracy of 91.98% in identifying cars driving incorrectly. Our system is different from this system in many ways: Our primary focus is on detecting wrong-turn violations at roundabouts, which can cause traffic deadlocks and increase the risk of collisions. In addition, our system employs a deep learning algorithm, with an expected initial accuracy of 80–90%.

Bedruz et al. (2018) proposed a robotic model technique to develop a trial traffic surveillance detection system, with an arrest script. The model includes two robots: One to track the moving object detected by the camera, and another to follow it if its speed exceeds a predetermined threshold. The camera’s recorded images are fed into an algorithm that determines whether an object is traveling faster than the reference speed by detecting the moving object and tracking its speed. When the object in motion exceeds the speed limit, the chaser robot is activated and follows it based on the algorithm’s output.

The deep learning-based car violation detection system developed by Xu et al. (2021) includes object detection, multi-object tracking, license plate identification, and multi-attribute recognition. Two additional modules for detecting red light running and improper pedestrian behavior are also integrated into the system. The system was tested at multiple traffic crossings, and the findings revealed that it can effectively track and detect cars, recognize number plates, and identify a range of violations, including red light running and improper pedestrian conduct. The system provides real-time traffic scene monitoring and data processing, which can help in smart traffic management. The results indicate that the recommended method is more efficient and affordable than standard detection and monitoring strategies based on physical infrastructure. The outcome of our proposed system is different from this because our system analyzes roundabouts through cameras and detects violations that occur at roundabouts.

The field of intelligent transportation systems (ITSs) leverages technology to improve transportation safety and efficiency. In studies conducted by Khanna et al. (2019), Arnob et al. (2020), Waris et al. (2022), and Ait Ouallane et al. (2022), the ITS is enhanced using deep learning algorithms to detect lane-based violations and helmet violations by motorcyclists. The enhanced systems are simulated, and their data transmission, performance, accuracy, prediction, and path-change planning are analyzed in detail.

The use of deep learning to recognize three-wheeled vehicles and detect violations of one-way traffic rules was suggested by Mampilayil and Rahamathullah (2019). The system uses the vehicle’s trajectory points to determine the direction of travel. A rule violation is recorded if a vehicle travels opposite to the designated one-way traffic direction. The proposed method is cost-effective and easy to deploy, as it does not require any sensors. This technique can be effectively used to manage traffic on roads, particularly in areas with one-way streets.

Azimjonov et al. (2023) and Rahman et al. (2020) proposed systems that detect vehicles under various weather conditions, including haze, dust, sandstorms, snow, and rain, both during the day and at night, using the YOLO machine learning algorithm.

Tang et al. (2022) discussed three aspects of automatic number plate recognition (ANPR). The primary aspect focuses on the technical development of ANPR technology. The secondary aspect examines the factors influencing its recognition performance in different contexts, while the tertiary aspect considers the use cases of this technology and its practical implications from a user’s point. It further discusses and identifies important underdeveloped areas, emerging themes, and policy similarities.

This section has highlighted significant advancements in deep learning techniques and their applications in traffic surveillance while also identifying notable gaps in existing research, particularly concerning the detection of wrong-turn violations at roundabouts. On the basis of these insights, Section 3 outlines the proposed methodology for addressing this specific issue. By developing a dedicated dataset and employing tailored deep learning models, the research aims to provide an effective solution for accurately detecting wrong-turn violations, thereby enhancing traffic management and road safety.

## 3 Proposed methodology

A machine learning-based approach for detecting wrong-turn traffic violations at roundabouts is presented in this section. The primary goal is to enhance the safety of roundabouts for all types of road users. Traffic engineers are expected to benefit from this research by managing these violations, thereby improving the overall experience at roundabouts. The proposed approach is based on the recommendation to strategically install cameras that capture the region of interest (ROI) where cars are involved in wrong-turn violations. The video footage will serve as input to a deep learning-based model for detecting violations. The deep learning model will be trained using our specially developed dataset.

The lack of a dedicated dataset for wrong-turn violations at roundabouts underscores the need for research in this area. By training a deep learning model specifically tailored for wrong turns at circular intersections, researchers can explore innovative solutions and test algorithms capable of accurately identifying such violations.

At roundabouts, there are four key points (Zutobi Drivers Ed, 2024) where wrong turns can be detected using static cameras and deep learning techniques. The cameras will evaluate whether a vehicle is making a wrong turn, and if a violation is detected, the system will label the vehicle accordingly.

**Figure 4:**
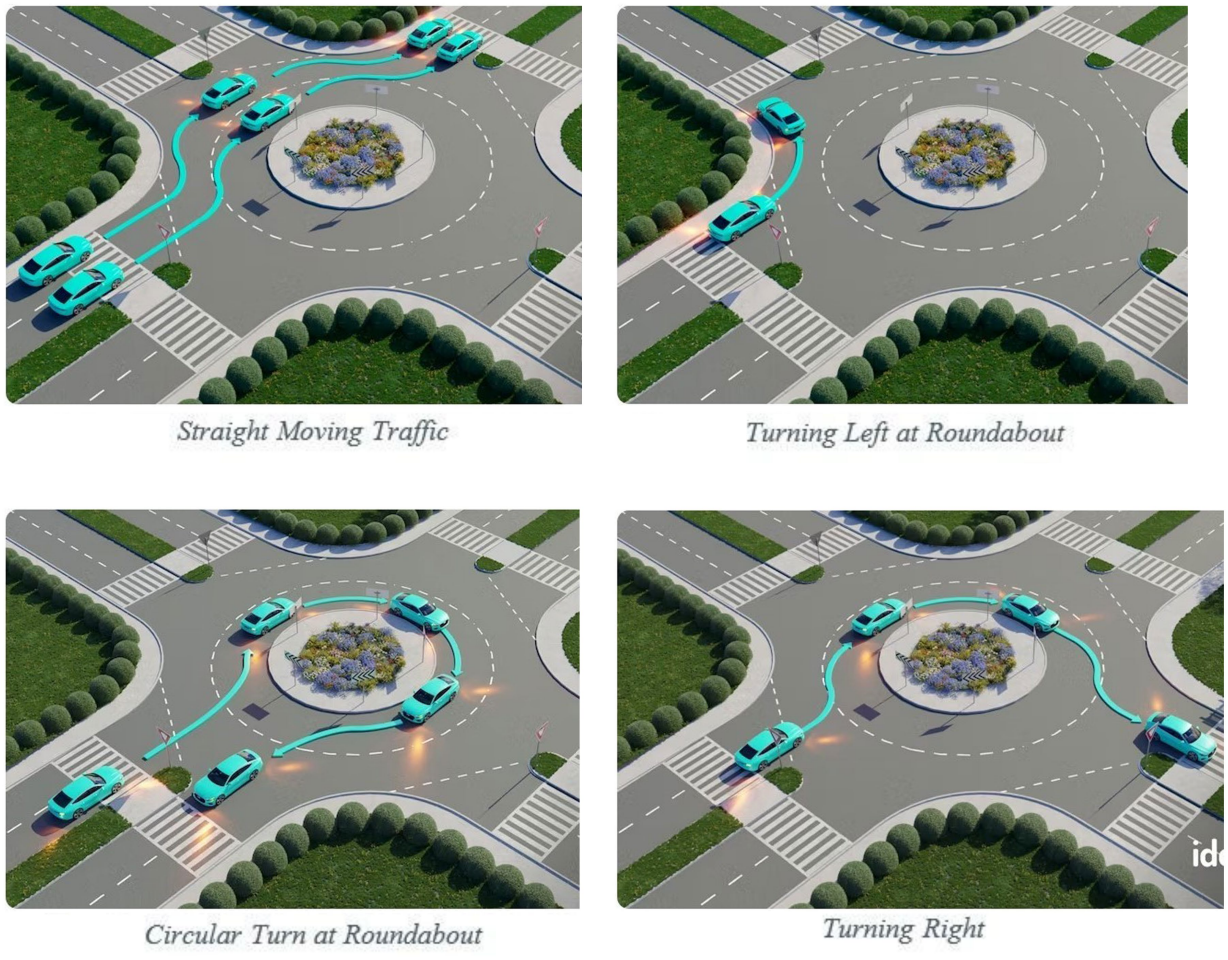
Possible traffic flows at a roundabout.

The proposed solution is based on the traffic violation scenario shown in Figure 2. It aims to reduce traffic hazards at roundabouts. In a roundabout, there are four possible turns: left, right, U-turn, and straight, as shown in Figure 4.

In three of these scenarios, human error is possible but wrong turns are not.

**Figure 5:**
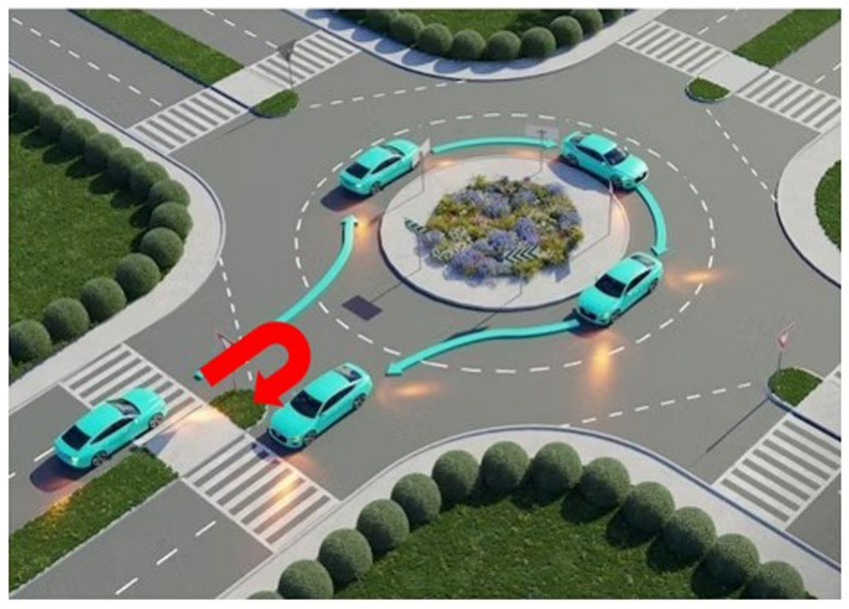
Expected wrong turn at a roundabout.

However, in the third scenario (Figure 4), a wrong turn can occur, which creates a hazard at the exit for vehicles that are either going straight or making a proper U-turn, as illustrated in Figure 5.

In some regions, such as Pakistan, it has been observed that drivers sometimes make improper U-turns at roundabouts, as shown in Figure 2. This behavior creates hazards for vehicles that are exiting the roundabout correctly, whether they are going straight or making a proper U-turn. To prevent these wrong turns, native solutions are sometimes implemented, which reduce wrong turns but also restrict the paths available to vehicles moving correctly, as shown in Figure 3.

This research aims to help authorities identify wrong turns, allowing them to avoid restrictive native solutions and keep the path open for vehicles following the rules. Our system detects vehicles making wrong turns, enabling authorities to take appropriate actions—such as issuing fines or legal notices—against the drivers responsible.

### 3.1 Proposed approach

#### 3.1.1 WorkFlow

**Figure 6:**
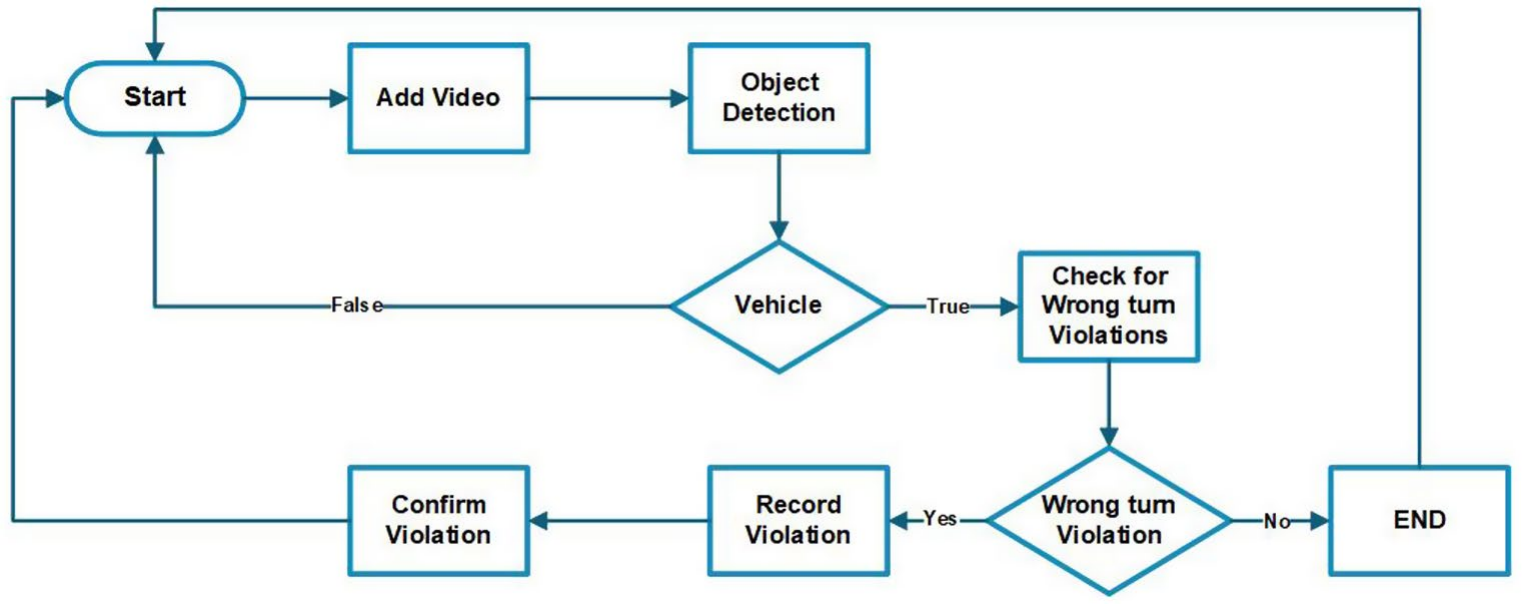
Basic workflow.

This workflow is designed to automate the detection and documentation of wrong-turn violations, providing an efficient and systematic approach to traffic monitoring. Figure 6 outlines the process of detecting and handling wrong-turn violations using video input. A step-by-step explanation is provided below:

1. *Start*: The process of detecting wrong turns starts here.
2. *Add video*: This step involves capturing video of the region of interest at the roundabout and providing it as input to the system.
3. *Object detection*: After the video is uploaded, the system uses object detection techniques to identify vehicles in the video.
4. Vehicle Detected?

*True*: If a vehicle is detected in the video, the system proceeds to the next step.
*False*: If no vehicle is detected in the video, the system loops back to the start of the process.

*Check for wrong-turn violations*: The system checks whether the detected vehicle has made a wrong turn.
Wrong-turn violation?

Yes: If a wrong-turn violation is detected in the video, the system proceeds to the next step to record the violation.
No: If no violation is detected in the video, the process will end.

*Record violation*: The system records the details of the wrong-turn violation.
*Confirm violation*: The system confirms the recorded violation to ensure the accuracy of the data.
End: The process concludes after confirming the violation or determining that no violation has occurred.

#### 3.1.2 Detection process

**Figure 7:**
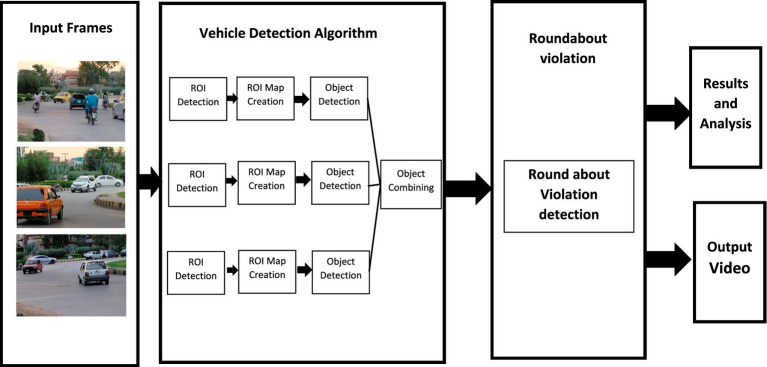
Block diagram.

Figure 7 elaborates on the vehicle and violation detection process. This diagram focuses on monitoring roundabouts. The different components of the process shown in the diagram are briefly explained below.

1. *Input frames*: The process begins with input frames, which are video frames capturing traffic at a roundabout. These images show different moving vehicles within the roundabout.
2. *Vehicle detection algorithm*: The creation of the ROI map involves manually outlining the roundabout roadway in the video frame using boundaries to exclude irrelevant areas such as sidewalks, buildings, the sky, and background. This ROI is defined once for each camera view and is used during detection and tracking so that only vehicles moving within the roundabout area are analyzed, which reduces false detections and enables reliable identification of wrong-turn movements based on vehicle trajectories. The input frames are fed into a vehicle detection algorithm. This algorithm operates in the following stages:

*ROI detection*: The algorithm detects the Region of Interest (ROI) in each frame where vehicles are likely to be present.
*ROI map creation*: After detecting the ROI, a map of these regions is created for further analysis.
*Object detection*: In this stage, the algorithm identifies and classifies objects within the ROI, typically focusing on detecting vehicles.
*Object combining*: This step combines detected objects to consolidate information across multiple frames or regions.

*Roundabout violation*: Once vehicles are detected and tracked through the algorithm, the system checks for any roundabout violations.
*Roundabout violation detection*: This process involves analyzing the vehicle’s movements to determine if any traffic rules related to roundabout navigation have been violated.
*Output and analysis*: The results of the violation detection process are then divided into two components:

*Results and analysis*: The detected violations and other relevant data are compiled for analysis, which could be used for reporting or decision-making.
*Output video*: A video is generated as an output, likely highlighting the detected violations for further review.

### 3.2 Evaluation methodology

#### 3.2.1 Data collection

The first phase of the research involved the creation of an image dataset, which was later annotated as part of the dataset preparation. During this phase, images were captured at locations where wrong-turn violations occurred. This involved capturing images of both violations and normal vehicle movements. A total of three roundabouts in the Hayatabad area of Peshawar were selected for image capture. Images were captured using high-quality smartphone cameras. The camera placement and the region of interest were determined according to the proposed approach. During image collection at specific locations to capture wrong-turn violations at roundabouts, a simple smartphone was used. Using this smartphone, various types of traffic wrong-turn violations were recorded. These violations were captured during both busy and quiet periods, as well as on weekdays and weekends. During this activity, ethical standards and privacy laws were closely observed to protect the privacy of individuals. While capturing the required scenes, measures were taken to avoid any interference with the normal traffic flow. This method of image collection was necessary both to record violations and to support better planning for the improvement of road safety in urban areas.

#### 3.2.2 Data annotation

Images in any dataset require proper annotations with labels or categories to be properly used for training machine learning models. In the second phase, we annotated the collected images. Computer Vision Annotation Tool (CVAT) was used for the annotation process. CVAT is a web-based application with a user-friendly interface. It provides various annotation options, such as bounding boxes, polygons, key points, and segmentation masks, to mark the region of interest (ROI). The annotation process involved sorting instances of wrong turns and traffic violations. To confirm consistency, accuracy, and reliability, predefined steps were followed. The images captured during the data collection process were uploaded to CVAT.

In CVAT, images were annotated using the suitable option. In the context of wrong turns at roundabouts, we used bounding boxes to outline traffic violations in the images. For this purpose, we carefully reviewed each image, and after careful review, we applied annotations to the images to accurately identify wrong turns and traffic violations. Throughout this repetitive process, consistency and focus of the user or annotator were essential to achieve accurate results.

During the annotation process, quality control steps were followed to ensure targeted accuracy. These steps included cross-validation by multiple annotators and review of the annotated images by project supervisors. Validation against ground truth data was also incorporated as part of the quality control process.

The annotated images were documented and stored. These records included metadata and annotation guidelines for future use. Such documentation helps maintain transparency and reusability in the research process.

The process of image annotation plays a vital role in converting images into a usable, labeled dataset. Such a dataset is a key requirement for training machine learning models and for conducting subsequent analyses. Researchers can produce high-quality datasets using CVAT. However, for a high-quality dataset, a step-by-step mandatory annotation procedure is also required. Following these steps, information is extracted from images for use in traffic analysis and research.

#### 3.2.3 Dataset analysis

The annotated images used in this study are key components for model training and analysis. This model was then evaluated for its performance at roundabouts where wrong turns frequently occur. The model was trained using the annotated dataset collected from local roundabouts in Peshawar, Pakistan. The dataset contained images from various conditions involving wrong turns. These images enabled the model to learn and recognize patterns associated with wrong turns.

It was possible to extract key information about landscape representation and the generality of traffic conditions from the annotated images. These insights could be obtained by evaluating the ROIs in the annotated images. During the evaluation process, the dataset was analyzed to assess traffic volume, the frequency of violations, and traffic jams at multiple locations. This allowed researchers to analyze and compare different roundabouts based on congestion and the frequency of violations. Using this information, data for each roundabout can be quantified to provide recommendations for traffic management and improvements. Researchers can study areas based on factual data and prioritize regions with frequent violations. These interventions may include campaigns to promote traffic rules and regulations, improvements to infrastructure, or other public awareness activities. Once researchers have a better understanding of the images across the study areas, they will be able to understand wrong-turn violations. In this study, all key points are focused on highlighting the study area. These points are intended to reduce traffic violation occurrence and improve the outcomes of programs designed to enhance road safety.

The composition of the annotated dataset can be described using facts and figures derived from the annotations made during this research. These statistics provide insights into the behavior of the dataset. The annotation statistics often focus on the types and frequency of traffic violations. These violations are also captured in the images. This research focused on analyzing these data to understand how often wrong-turn violations occur. This research examined how annotations are distributed across different types of violations. It further aimed to train and evaluate the model on the most common and serious traffic wrong-turn violations.

This research also examined metrics derived from the annotation statistics to assess the quality and reliability of the dataset. This quality check is based on measures such as annotation density and annotation completeness. Notably, a high annotation density indicates that the dataset provides a detailed representation of traffic wrong-turn violations. High inter-annotator agreement indicates that annotators are consistent in identifying traffic wrong turns. These metrics are critical for assessing the strength and utility of the dataset for training and testing deep learning models. In addition, the research provides guidance for improving and validating the dataset as the study progresses.

#### 3.2.4 Model training

In this phase, a deep learning model was trained using a newly created and annotated dataset. PyCharm was selected as the development environment. PyCharm offers a powerful and adaptable development environment that meets the requirements for building and training deep learning models for traffic analysis and violation detection.

Annotated images are fed into the deep learning model for training. The training process teaches a deep learning model to recognize and classify wrong turns and traffic violations. The training process basically follows supervision-based learning approaches. In supervised learning, the model is trained on the dataset of annotated images.

A total of 2,700 images were extracted and manually annotated. The dataset was divided into 70% for training, 15% for validation, and 15% for testing. Validation was performed during training using the separate validation subset comprising 15% of the annotated images to prevent overfitting. During training, the model was evaluated on this validation set at the end of each epoch to monitor performance metrics, such as precision, recall, and loss.

The model periodically changed its parameters and scales on the annotated images during the training process. It also changed loss functions to improve accuracy and reduce errors, such as prediction errors. Training involved many epochs, with the dataset provided to the model in segments. Throughout training, many procedures, including backpropagation, were used to change the model’s parameters. Parameters such as segment size, model sampling frequency, and tuning settings were carefully adjusted to improve model performance.

#### 3.2.5 Model evaluation

In the next phase, we evaluated the performance of the model trained on our dataset. A separate dataset was used to evaluate model performance. During evaluation, the unused dataset was fed into the model for testing purposes. Standard evaluation metrics such as accuracy, F1-score, and recall were calculated to assess model performance. The F1-score is computed as shown in Equation (1).


$$F1\;\;\text{Score}=\frac{2(\text{Precision}\times \text{Recall})}{\text{Precision}+\text{Recall}}$$ (1)

Evaluation was carried out based on the model’s recognition ability and the actual dataset used during training. In this evaluation step, confusion matrices were also used to assess the strengths and weaknesses of the trained model. The limitations revealed during evaluation highlight areas for improvement. The evaluation results provided insights for refining and optimizing the model’s performance, which can enhance its effectiveness in real-world scenarios. The experiments were conducted on a dataset of 2,700 annotated images, divided into 70% for training, 15% for validation, and 15% for testing. The model was trained for *57* epochs using standard YOLO hyperparameters, and validation was performed after each epoch to monitor convergence and avoid overfitting. Performance was evaluated on the test set using precision, recall, and mAP@0.5 metrics.

## 4 Results

This section presents the findings of the research, which focus on detecting wrong-turn violations using deep learning techniques. The primary goal of the study is to address a research gap by creating a dataset and assessing the performance of a deep learning model in identifying wrong-turn violations at roundabouts using the locally created dataset. The results are based on an evaluation of the trained model’s ability to recognize wrong turns at roundabouts. The evaluation included accuracy, precision, and recall to examine the model’s performance in scenarios such as wrong-turn violations at roundabouts. The results are based on the information obtained from the evaluation of the trained model. They showed that the model performed well in detecting wrong-turn violations at roundabouts, with an accuracy of 86%.

### 4.1 Visualization of wrong turns

**Figure 8:**
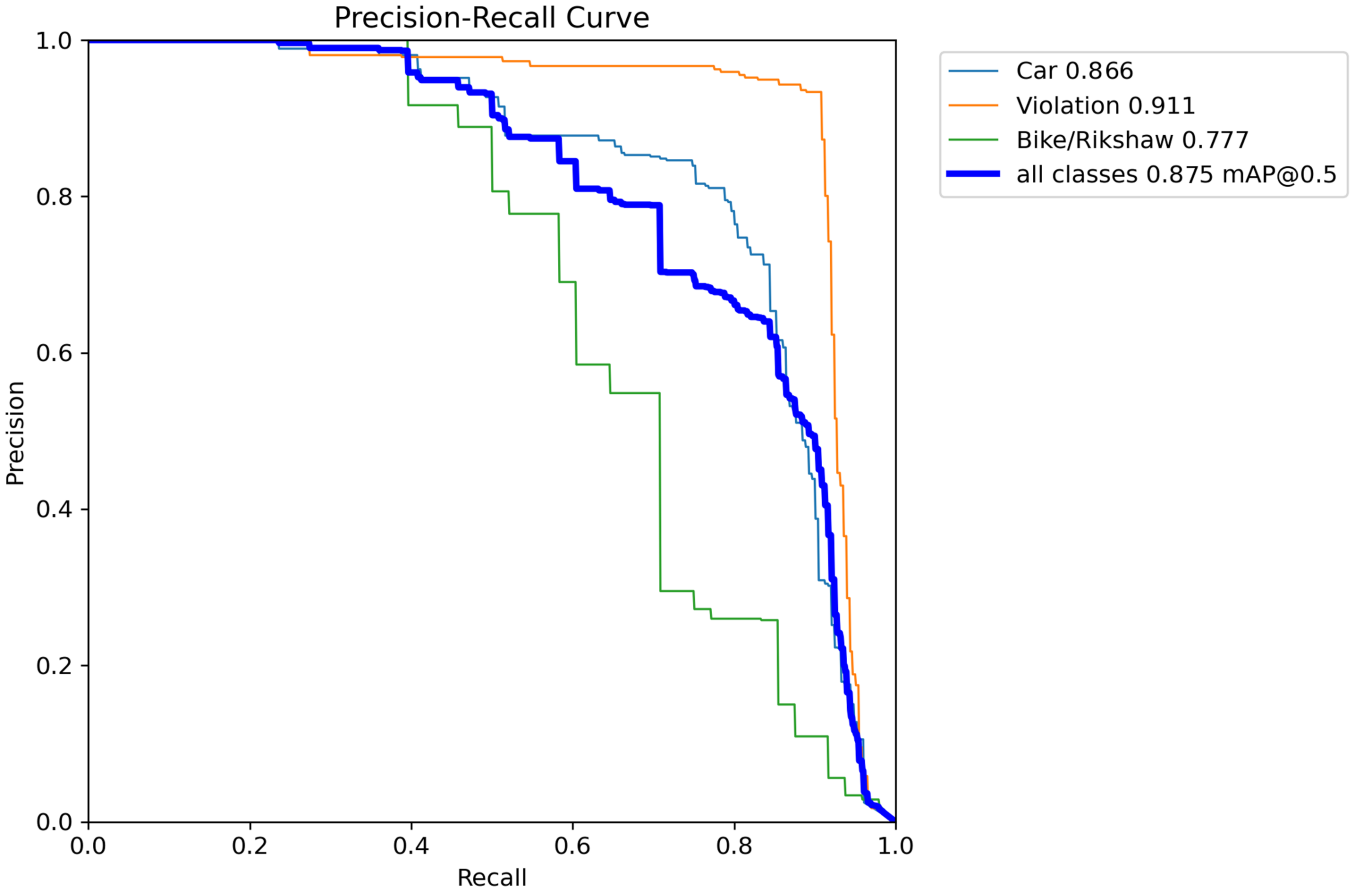
Precision–recall curve.

This section provides a comprehensive summary of the model’s performance in recognizing wrong-turn violations at roundabouts, as illustrated in the annotated image (Figure 3). The results are highlighted through visual representations of wrong turns. For this purpose, different types of visuals were used to aid in the identification of wrong-turn violations. These visuals included precision–recall curves, confidence–precision curves, and recall–confidence curves to evaluate the levels of accuracy. The detection categories included wrong-turn violations at roundabouts for cars and bikes/rickshaws. Figure 8 illustrates all the considered categories.

**Figure 9:**
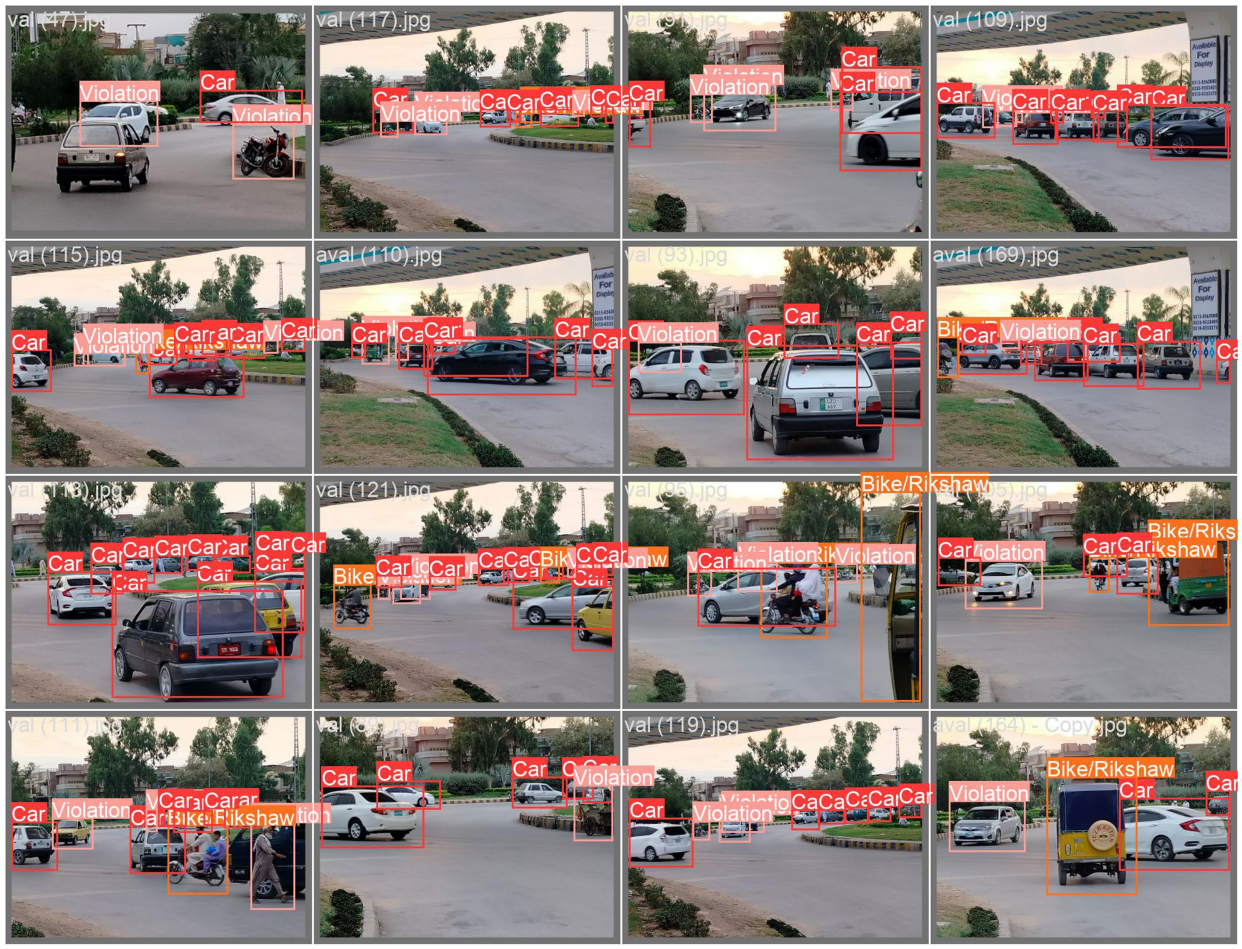
Visualizations of wrong-turn violation detections.

Precision–recall curves display the trade-off between precision and recall at different confidence levels. This trade-off shows the model’s capability to accurately recognize wrong-turn violations at roundabouts across varying thresholds. Similarly, confidence–precision curves illustrate the precision of the model’s predictions at different levels. These curves show its confidence in detecting objects, such as wrong-turn violations for cars and bikes/rickshaws, as shown in Figure 9.

Recall–confidence curves show the extent to which the model remembers wrong-turn violations at different confidence levels. This helps the observer understand how active the model is. The model’s effective performance depends on correctly identifying true positives while minimizing false negatives. It is clear that these visualizations provide a clear sense of the model’s performance and help stakeholders evaluate its effectiveness in real-world situations.

### 4.2 Comparative analysis of study areas

The comparative analysis in this research examined the prevalence of wrong-turn violations, their frequency of occurrence, and the extent to which they are recognized as violations at roundabouts. This analysis also enabled the identification of patterns, trends, and differences related to road safety and traffic management.

The violation rate is a statistical term that can be calculated and compared across studies. This comparison aimed to quantify the relationship between this research and traffic regulations and enforcement measures. In addition, stakeholder interviews may enhance the quantitative analysis by providing context and insights into the factors that promote wrong-turn behaviors.

**Figure 10:**
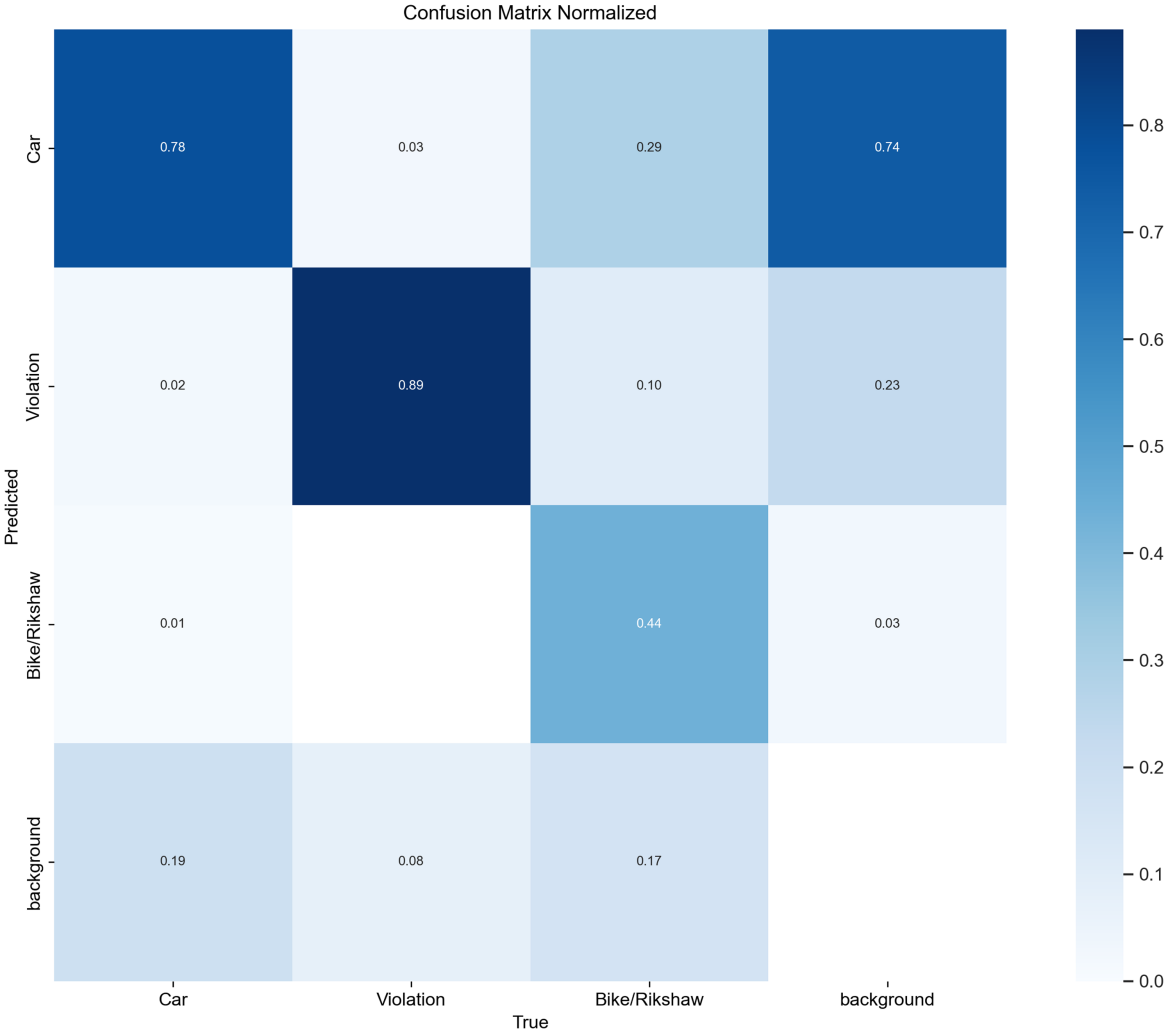
Normalized confusion matrix.

The comparative analysis also highlighted differences in infrastructure design, traffic flow patterns, enforcement strategies, and socio-economic factors that contribute to wrong-turn violations, as shown in Figure 10. These insights are valuable for policymakers, urban planners, and traffic authorities in allocating resources and implementing targeted measures to minimize the risks associated with wrong-turn violations at high-traffic roundabouts.

**Figure 11:**
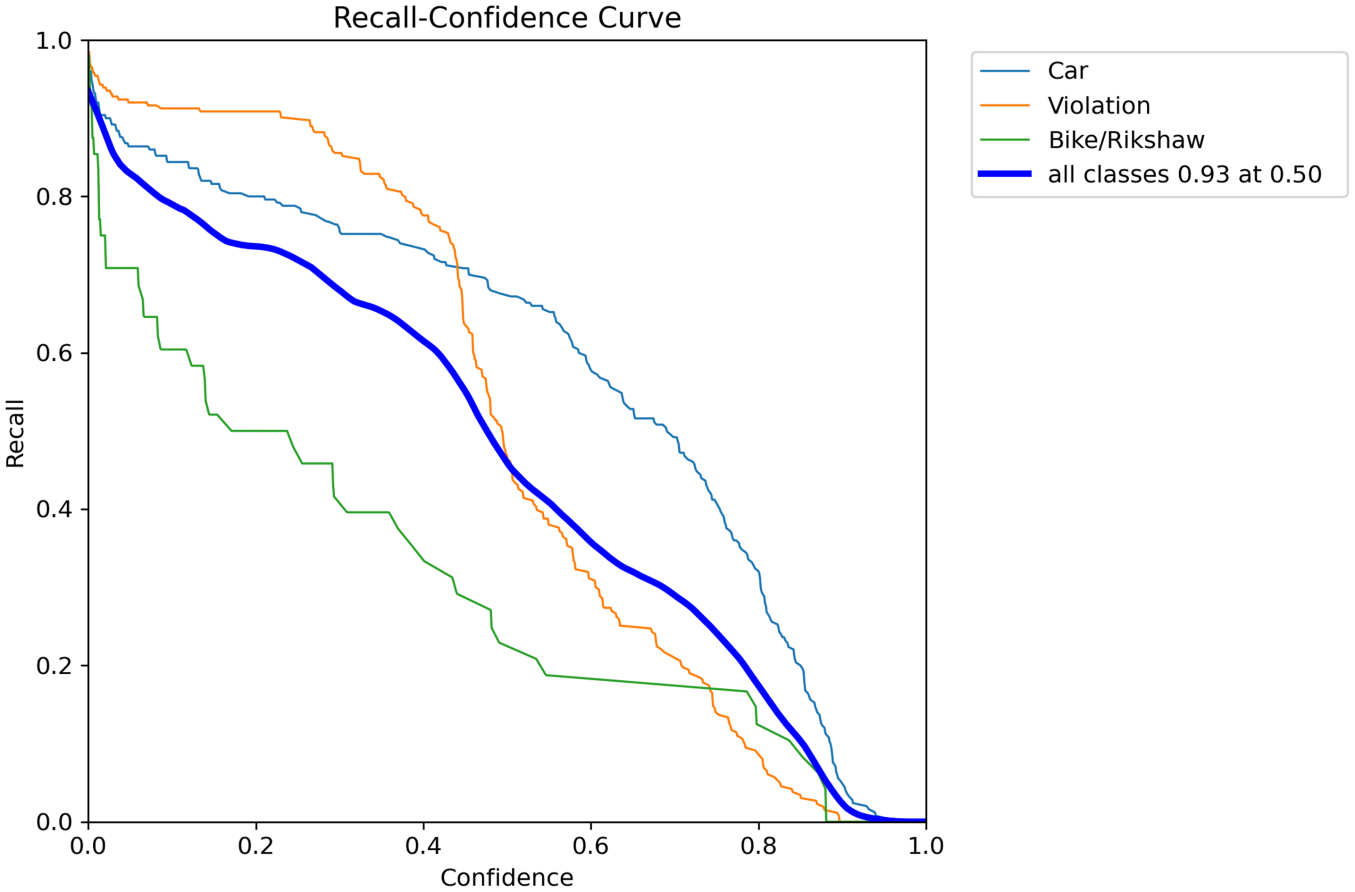
Recall–confidence curve.

Across different classes, such as cars and bikes/rickshaws, varying prediction accuracies were observed. Notably, the model achieved an accuracy of 89% in identifying wrong-turn violations. The confusion matrix was used to analyze misclassifications among the labeled object classes, while the background class, which represents non-object regions, was not considered for the accuracy evaluation, as highlighted in Figure 11. This indicates that the model demonstrated impressive accuracy in predicting instances labeled as violations. This means that when the model encountered wrong-turn violations, it correctly identified them with an 89% success rate. This accuracy was crucial for the model’s performance evaluation.


$$\text{Accuracy}=\frac{\mathrm{TP}+\mathrm{TN}}{\mathrm{TP}+\mathrm{TN}+\mathrm{FP}+\mathrm{FN}}$$ (2)

**Figure 12:**
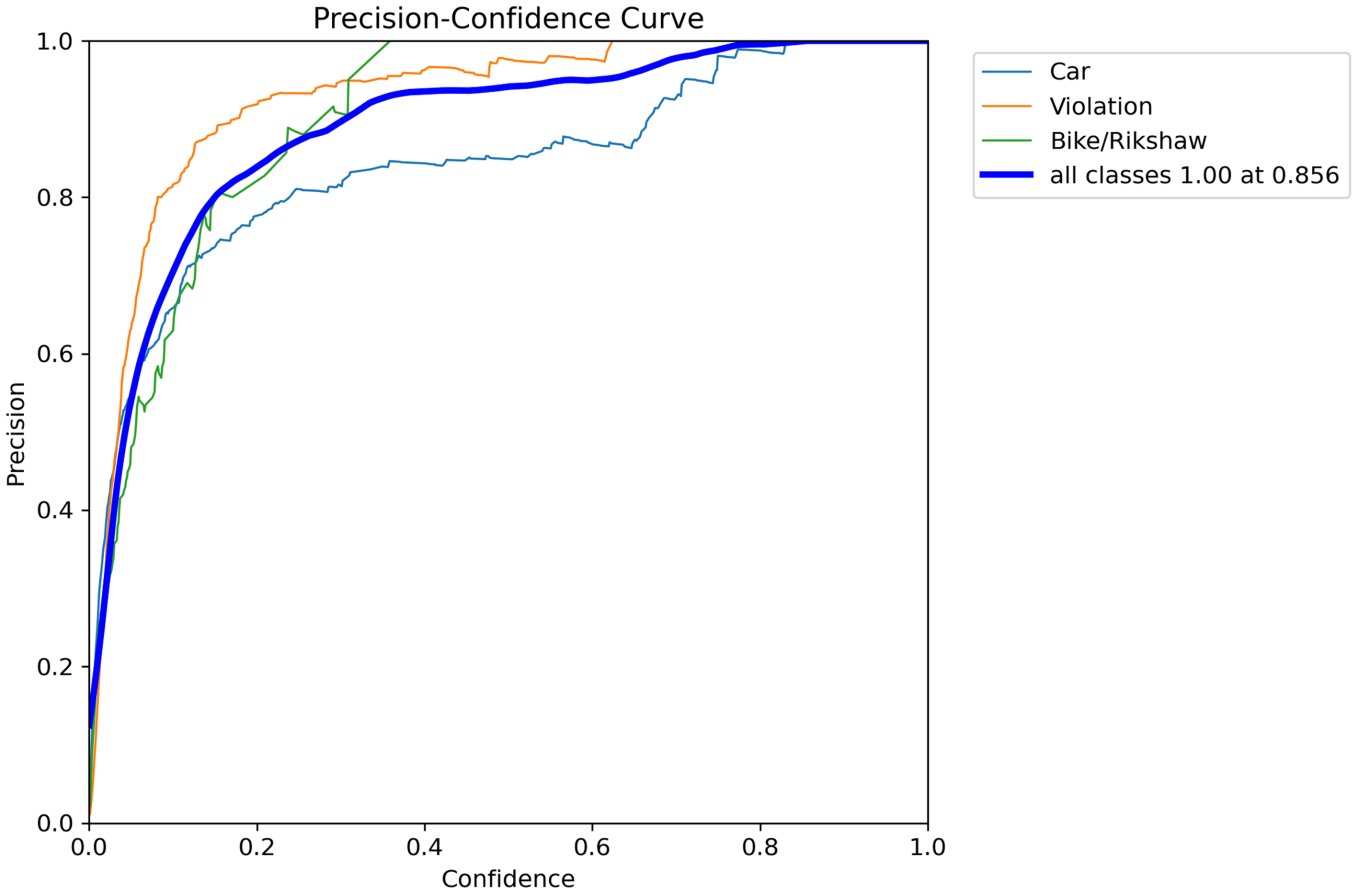
Precision–confidence curve.

In Equation 2, TN represents true negatives, TP represents true positives, FP represents false positives, and FN represents false negatives. The model also showed good accuracy in identifying cars, with a precision of 0.85 at the specified confidence level. Accuracy was critical for the application of the model. The individual curves represent the precision–recall performance for each object class (Car, Violation, and Bike/Rickshaw) computed on the test set. The “all classes” curve represents the aggregated performance across all classes, obtained by averaging precision over all classes at different recall levels. The corresponding value reported for the “all classes” curve reflects the mean average precision (mAP@0.5), which summarizes overall detection performance rather than class-specific behavior. In Figure 12, the orange line represents the violation class. The model performed with a precision of 85.6% and could effectively detect wrong-turn violations. The model also included bike/rickshaw predictions. The accuracy curve exhibits a pattern similar to that of violations, but with lower values. The annotation of all classes (1.00) at 0.856 represented optimal precision. At this combined confidence level, the model showed flawless precision for all classes, as shown in Figure 12. The value 0.856 was not arbitrarily selected as a confidence threshold; rather, it represents the empirically determined optimal confidence threshold at which the model achieved the best precision performance on the validation set. This value was obtained by sweeping the confidence threshold from 0 to 1 and identifying the point at which the aggregated precision across all classes reached its maximum. The precision metric used to evaluate the model performance is calculated as shown in Equation (3).


$$\text{Precision}=\frac{\mathrm{TP}}{(\mathrm{TP}+\mathrm{TF})\times \text{Recall}}=\frac{\mathrm{TP}}{\mathrm{TP}+\mathrm{FN}}$$ (3)

The model obtained an overall precision of 0.86. When identifying a car, it was 86.6% accurate. High precision was required to eliminate false positives. The model performed well in the violation class, achieving a precision of 0.911 (Figure 8). When detecting a violation, it was 91.1% accurate. Precision was critical for the application of the model. The model predicted bike/rickshaw instances with a precision of 0.777. Overall, the aggregate evaluation of all classes (shown by the dark blue line) produced a mean average precision (mAP@0.5) of 0.875. Figure 8 provides a detailed overview of the model’s performance statistics.

The model showed a high TP rate for wrong-turn violations. When the model recognized a violation, it correctly recalled it 93% of the time. It performed well in the violation class. Recall is necessary for application in security and law enforcement. The model performed slightly worse for bike/rickshaw predictions, where recall was relatively lower. Overall, the aggregate evaluation across all classes produced a recall of 93% at a confidence level of 0.5, providing a complete picture of the model’s performance, as highlighted in Figure 11.

**Figure 13:**
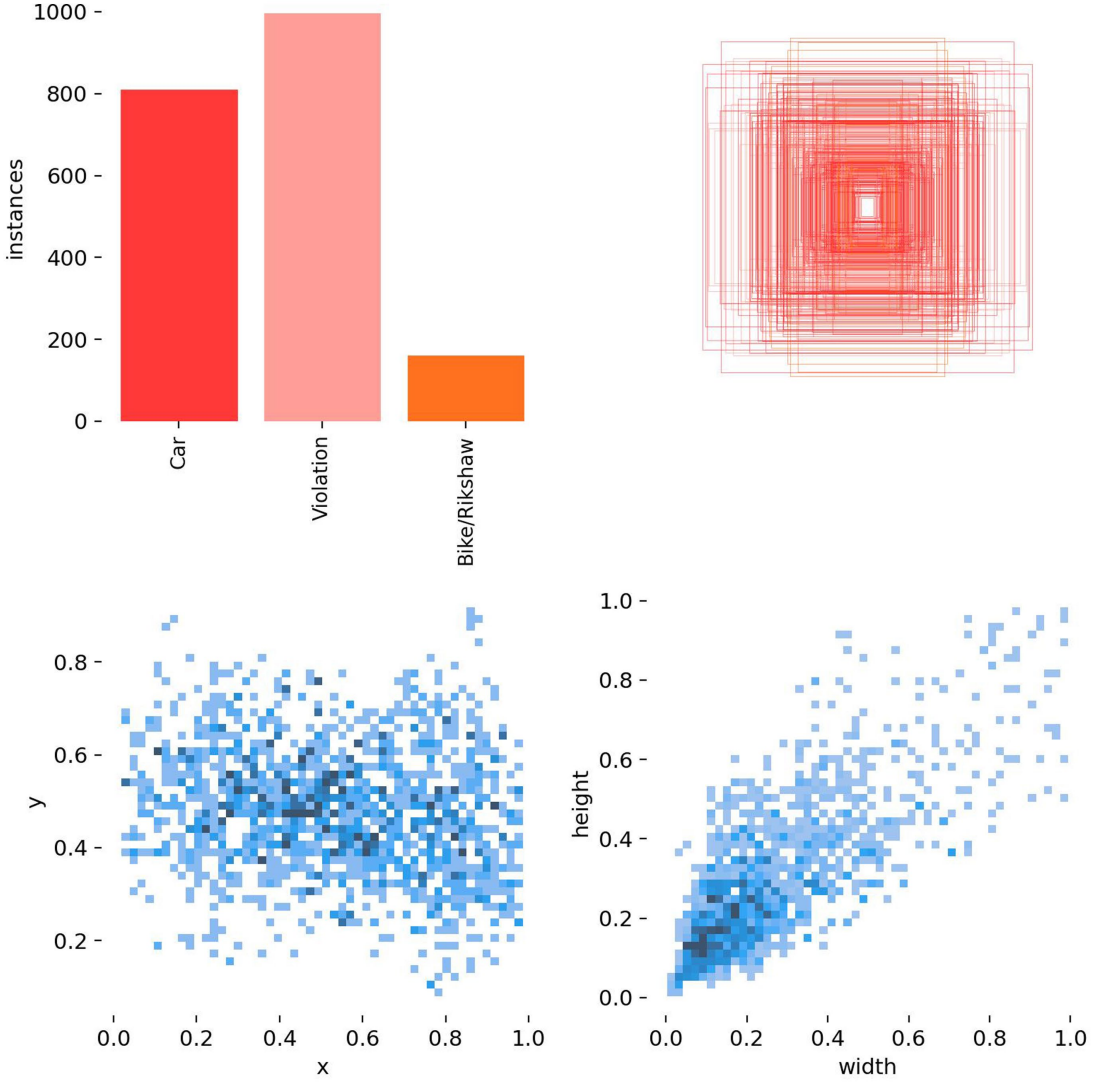
Relationship between variables.

The bar graph in Figure 13 displays the instance counts for three categories: Car, violation, and bike/rickshaw. Notably, the counts for violation and car were significantly higher than those for bike/rickshaw. This suggests that the dataset focused more on violations and cars due to their importance in the context of this research.

The red concentric pattern plot represents data density or frequency in a two-dimensional space. The increasing intensity toward the center indicates higher data concentration in that region. This pattern reveals underlying trends or clusters, showing that the data are concentrated in specific ROIs. The scatter plots, also known as 2D histograms, show correlations between different pairs of variables (x and y or width and height). The variables x and y represent the normalized center coordinates of the annotated bounding boxes within the image frame, with values ranging from 0 to 1 relative to the image width and height. The width and height represent the normalized dimensions of the bounding boxes, indicating the relative size of the detected objects in the dataset. In specific regions, the concentration of blue dots suggests patterns or clusters. The plots in Figure 13 provide insights into the relationships between the variables and support further analysis.

### 4.3 Discussion

#### 4.3.1 Interpretation of the results

The results of this study present an analysis of deep learning-based approaches for detecting wrong-turn violations. This study also discusses the efficiency of the model, which is based on a deep learning algorithm, in roundabout scenarios. The precision, recall, and accuracy metrics of this model enabled researchers to analyze its performance in identifying traffic violations. The targeted violations were wrong-turn violations. This research aimed to achieve a high level of accuracy. The precision–recall curves show the trade-off between precision and recall across different confidence levels. These insights provide a clear understanding of the model’s performance and allow researchers to effectively evaluate it. This model achieved high precision and recall rates for two classes: Wrong-turn violation and car. This indicates the effectiveness of the deep learning model in accurately identifying this type of traffic violations. At the same time, the recall rate for bike/rickshaw was relatively lower, due to the fact that the target was wrong-turn violation detection. These insights also suggest areas for further refinement and optimization of the model. Visualizations of wrong turns using bar graphs and scatter plots help show temporal patterns in traffic violations. In this research, these visualizations are used to identify hotspots of wrong-turn violations and to understand the distribution of traffic violations.

#### 4.3.2 Implications for traffic management

The results of this study have a significant impact on traffic management practices. Traffic authorities can enhance their monitoring and enforcement capabilities using deep learning techniques for real-time detection of wrong-turn violations. This can improve the road safety system and reduce the number of traffic crashes. The creation of a local dataset for a deep learning-based wrong-turn violation detection system, intended to replace the native solution, is the primary outcome of this research. Concerned authorities can detect wrong-turn violations and other violations using intelligent cameras. The deployment of these cameras can help ensure safer roundabouts for drivers.

This study was carefully conducted to ensure the reliability of the dataset and model. To highlight the importance of data-based techniques in managing traffic violations, images were collected from diverse areas. This collection will help policymakers and other decision-makers in making informed decisions. This research also identifies areas for future research that could further improve road safety. This repetitive procedure of image capturing, annotating, and analyzing will enable decision-makers to make informed decisions.

#### 4.3.3 Comparison with native techniques

In comparison with the proposed deep learning–based approach, traditional methods for addressing wrong turns often rely on physical measures such as placing stones or barriers at roundabouts, as illustrated in Figure 4. Although these conventional solutions aim to prevent wrong-turn violations, they are typically dependent on physical infrastructure and often lack adaptability and scalability. These native solutions are not practical in rapidly changing urban traffic environments because, in cities, traffic directions and patterns change frequently for many reasons. In addition, these native solutions require regular maintenance and are costly to install and implement. In comparison, the advanced solution proposed in this study, based on a deep learning approach, offers a flexible, cost-effective, and scalable alternative. This solution is designed to detect wrong-turn violations in real time. Models capable of identifying wrong-turn violations can be developed by training deep learning algorithms on a dedicated dataset. These models can be deployed in violation detection systems. More broadly, violation detection systems could take the form of mobile applications or other traffic management platforms to provide timely alerts to stakeholders. This enables proactive actions and assists the relevant authorities in enforcing traffic laws.

## 5 Conclusion

This article addresses an often-overlooked yet important traffic issue: Wrong-turn violations at roundabouts. A wrong-turn violation at a roundabout occurs when a vehicle, instead of completing the full circle and exiting properly, makes an illegal shortcut or U-turn without navigating the roundabout correctly. This is a highly hazardous driving behavior. This problem is especially prevalent in large roundabouts in many developing cities. In this study, we proposed an end-to-end solution based on a deep learning approach to tackle this issue. We validated the approach using a YOLO-based deep learning model. Due to the absence of an existing image dataset for this purpose, we created and manually annotated a local dataset featuring roundabouts in Peshawar, Pakistan. The model achieved high precision and recall on unseen images of wrong-turn violations, demonstrating its ability to detect such events in real time and under natural urban conditions. Our contributions are threefold. First, we present the first publicly available dataset focused specifically on wrong-turn behavior at roundabouts. Such a dataset can help other researchers advance this area of research. Second, we propose a deep learning-based method for detecting these violations. Third, we demonstrate that a fine-tuned single-stage detector can achieve field-ready performance without relying on expensive hardware. Such automated violation detection can reduce reliance on manual monitoring and physical barricades, lower enforcement costs, and enhance road safety for drivers, cyclists, and pedestrians alike. Furthermore, such an approach can contribute to the broader adoption of intelligent, data-driven traffic management systems.

## Data Availability

The dataset used in this study is publicly available on Figshare and can be accessed at: https://doi.org/10.6084/m9.figshare.31572715.
